# Comparison of rumen bacterial communities in dairy herds of different production

**DOI:** 10.1186/s12866-017-1098-z

**Published:** 2017-08-30

**Authors:** Nagaraju Indugu, Bonnie Vecchiarelli, Linda D. Baker, James D. Ferguson, Jairam K. P. Vanamala, Dipti W. Pitta

**Affiliations:** 1 Department of Clinical Studies, School of Veterinary Medicine, New Bolton Center, University of Pennsylvania, Kennett Square, PA 19348 USA; 2 Department of Food Science, Pennsylvania State University, University Park, State College, PA 16802 USA; 3 Penn State Hershey Cancer Institute, Hershey, PA 17033 USA

**Keywords:** Dairy cows, Rumen microbiota, Dairy herds

## Abstract

**Background:**

The purpose of this study was to compare the rumen bacterial composition in high and low yielding dairy cows within and between two dairy herds. Eighty five Holstein dairy cows in mid-lactation (79–179 days in milk) were selected from two farms: Farm 12 (M305 = 12,300 kg; *n* = 47; 24 primiparous cows, 23 multiparous cows) and Farm 9 (M305 = 9700 kg; *n* = 38; 19 primiparous cows, 19 multiparous cows). Each study cow was sampled once using the stomach tube method and processed for 16S rRNA gene amplicon sequencing using the Ion Torrent (PGM) platform.

**Results:**

Differences in bacterial communities between farms were greater (Adonis: R^2^ = 0.16; *p* < 0.001) than within farm. Five bacterial lineages, namely *Prevotella* (48–52%), unclassified Bacteroidales (10–12%), unclassified bacteria (5–8%), unclassified Succinivibrionaceae (1–7%) and unclassified Prevotellaceae (4–5%) were observed to differentiate the community clustering patterns among the two farms. A notable finding is the greater (*p* < 0.05) contribution of Succinivibrionaceae lineages in Farm 12 compared to Farm 9. Furthermore, in Farm 12, Succinivibrionaceae lineages were higher (*p* < 0.05) in the high yielding cows compared to the low yielding cows in both primiparous and multiparous groups. *Prevotella*, S24-7 and Succinivibrionaceae lineages were found in greater abundance on Farm 12 and were positively correlated with milk yield.

**Conclusions:**

Differences in rumen bacterial populations observed between the two farms can be attributed to dietary composition, particularly differences in forage type and proportion in the diets. A combination of corn silage and alfalfa silage may have contributed to the increased proportion of Proteobacteria in Farm 12. It was concluded that Farm 12 had a greater proportion of specialist bacteria that have the potential to enhance rumen fermentative digestion of feedstuffs to support higher milk yields.

**Electronic supplementary material:**

The online version of this article (10.1186/s12866-017-1098-z) contains supplementary material, which is available to authorized users.

## Background

Nearly 70% of energy [1] and 60–85% of protein [2] requirements of the dairy cow are met from microbial fermentation, indicating a critical need for maximizing rumen function and describing rumen microbiota. However, it is still not known how diet and microbes interact to enhance milk yields in dairy cows. Typically, dairy herds are fed total mixed rations (TMR) and cows with greater milk production have greater dry matter intake (DMI) [3]. In TMRs for dairy cattle, carbohydrates constitute nearly 70% of the dietary dry matter (DM) and provide the major energy source for rumen microbes [4, 5]. Carbohydrates may be broadly classified into two distinct groups based on their solubility in neutral detergent [6, 7]. Neutral detergent fiber (NDF) is insoluble in neutral detergent solution and is composed of cellulose, hemicellulose and lignin. Cellulose and hemicellulose are fiber carbohydrates (FC) predominantly found in forages. FC ferment slowly and are important in regulating rumen function through formation of the rumen mat and influencing the rate of passage out of the rumen. Non-Fiber Carbohydrates (NFC) are soluble in neutral detergent solution and include sugars, starch, beta-glucans and pectins, which ferment more rapidly in the rumen [7]. To maintain a stable rumen environment and enhance microbial growth, a minimum NDF and a maximum NFC (% diet DM) are needed in high producing dairy cow diets. Excessive NFC can create acidosis, while excessive NDF and low NFC can constrain feed intake and milk production [8]. The proportion of NDF and NFC in the diet, the composition of NFC components, and the extent to which these carbohydrates ferment in the rumen can influence the ruminal microbiota [9, 10].

Corn silage represents a major feed resource and often comprises 50% to 70% of forage in diets in the Northeastern US [11, 12]. Dairy One (Ithaca, NY), a commercial laboratory, reports that NDF averaged 44.2% DM (SD 5.3) and starch 30.7% DM (SD 6.5) in 11,281 corn silage samples analyzed between May 1, 2014 and April 30, 2015 (http://dairyone.com/2016-fresh-corn-silage-results-ny-and-pa/). Corn silage is classified as forage, however contains varying amounts of grain, contributing both dietary NDF and starch [8]. Concentrations of NDF and starch in corn silage are dependent upon plant genetics, environmental conditions, and maturity and processing of the corn grain at harvest [8] and can have a major impact on ration fermentability, and most likely the rumen microbiota. Lettat et al., [12] found cows fed a corn silage based TMR had an increase in total bacteria, an increase in propionate and a decrease in methane production compared to cows fed an alfalfa silage based TMR. However, information concerning the influence of corn silage based diets on the microbial ecology in the rumen of dairy cows is limited.

Information on rumen microbial dynamics in dairy cows is emerging [13–16]. Recent reports include changes in the composition of ruminal microbiota in dairy cows in association with parity [15–17], diet [18, 19], breed [20, 21], feed efficiency [14, 15, 22], milk yield and composition [19, 23] and physiological status [16, 18, 24, 25]. The consensus of these reports indicates the preponderance of lineages from Bacteroidetes and Firmicutes among the rumen microbiota. However, it is not known which bacteria are relevant and in what proportions they are needed to enhance rumen fermentation of feedstuffs. In this study, our goal was to link how dietary components influence rumen bacterial populations, and how they may impact milk yield and composition in dairy cows. Here, we sampled rumen contents from primiparous and multiparous dairy cows selected from two dairy herds differing significantly in their average annual milk production. We analyzed the composition of rumen bacterial communities using Ion Torrent (PGM) sequencing and investigated their relationship with nutrition and production parameters.

## Methods

### Experimental details

This study included 85 animals of Holstein breed selected from two dairy herds in southeastern Pennsylvania, a higher producing farm (Farm 12; M305 = 12,300 kg; *n* = 47 including 24 primiparous, 23 multiparous cows (11 s, 4 third, 6 fourth, and 2 fifth lactation cows)) and a lower producing farm (Farm 9; M305 = 9700 kg; *n* = 38 including 19 primiparous, 19 multiparous cows (11 s, 6 third, and 2 fourth lactation cows). Production information including milk yield (kg/d), protein (%), and fat (%) for experimental cows were retrieved from Dairy Record Management Systems (DRMS). We differentiated cows into high and low milk production based on their previous milk test day results for each farm and parity group. As a result, we observed at least 9 kg difference in daily milk yield within each parity group. The selected cows were between 79 and 179 days in milk production (DIM) (Additional file 1: Table S1) with an average of 113 and 120 days for primiparous cows and 125 and 131 days for multiparous cows in Farm 12 and Farm 9, respectively. Dairy cows that were donors of rumen fluid were maintained according to the ethics committee and IACUC standards for the University of Pennsylvania (approval #805538).

TMR samples were collected immediately after presentation to the cows. Five samples were scooped from the dispersed feed at varying locations, combined into one sample and frozen at -20 °C. These feed samples were dried at 55 °C for 72 h and ground in a Wiley mill (Thomas Scientific, Swedesboro, NJ) using a 1-mm screen. DM, CP (Kjeldahl), ADF, sugars and ash were assayed according to AOAC [26]. The NDF was determined using sodium sulfite and a heat-stable α-amylase enzyme (A3306, Sigma-Aldrich, St. Louis, MO) according to the procedure of Van Soest et al. [27]. The starch content was determined using a commercial kit (Megazyme International Ireland Ltd., Bray, Ireland) based on the enzymatic method [23]. Mineral contents were analyzed by the atomic absorption spectroscopy method and protocol of AOAC [26].

### Rumen sampling

Rumen contents were sampled once from animals on each farm 2 h post-feeding using the stomach tube method [28]. The initial volume collected, approximately 200 mL, was discarded due to possible contamination with saliva and the subsequent 250 ml sample (planktonic phase) was obtained, transferred into 15 ml falcon tubes and snap frozen in liquid nitrogen. The samples were then transported to the laboratory and archived at −80 °C.

### DNA extraction, PCR and 16S rRNA gene sequencing

The genomic DNA was extracted from rumen samples using the PSP Spin Stool DNA Plus Kit (Invitek, Berlin, Germany). The DNA extraction method was adapted from Dollive et al. [29]. Briefly, the method involved taking 300 mg of sample in a Lysing Matrix E tube (MP BIomedicals, Solon, OH USA) and bead beaten in 1400 μl of stool stabilizer from the PSP kit to break open cell walls and release nucleic acid material. Samples were then heated at 95 °C for 15 min, placed on ice for 1 min, and spun down at 13,400 g for 1 min. The supernatant was then transferred to the PSP InviAdsorb tubes and the remainder of the protocol for the PSP Spin Stool DNA Plus was followed according to the manufacturer’s instructions. The genomic DNA was amplified using specific primers 27F and BSR357, targeting the V1–V2 region of the 16S rRNA bacterial gene. The forward primer carried the Ion Torrent trP1 (5′-CCTCTCTATGGGCAGTCGGTGAT-3′) and the reverse primer carried the A adapter (5′-CCATCTCATCCCTGCGTGTCTCCGACTCAG-3′), followed by a 10–12 nucleotide (nt) sample-specific barcode sequence and a GAT barcode adapter. The PCR mix was prepared using the Accuprime Taq DNA polymerase System (Invitrogen, Carlsbad, CA).

The thermal cycling conditions involved an initial denaturing step at 95 °C for 5 min followed by 25 cycles (denaturing at 95 °C for 30 s, annealing at 56 °C for 30 s, extension at 72 °C for 90 s) and, finally, an extension step at 72 °C for 8 min as described in Pitta et al. [18]. Amplicons of 16S rRNA genes were purified using 1:1 volume of Agencourt AmPure XP beads (Beckman-Coulter, Brea, CA, USA). The purified PCR products from the rumen samples were pooled to achieve a concentration of 5–20 ng prior to sequencing in Ion Torrent (PGM) platform.

### Data analysis

The 16S pyrosequence reads were analyzed using the QIIME pipeline [30], and in R 3.3.1 [31]. All sequences were quality filtered. Sequences shorter than 50 nt and longer than 480 nt, incorrect primer sequences, and those containing one or more ambiguous bases or homopolymers longer than 5 nt were discarded. Operational taxonomic units (OTUs) were formed at 97% similarity using UCLUST [32]. We randomly sub-sampled (rarified) the resulting OTUs to 3353 sequences per sample. Representative sequences from each OTU were aligned to 16S reference sequences with PyNAST [33] which were used to infer a phylogenetic tree with FastTree [34]. Taxonomic assignments within the GreenGenes taxonomy [35] were generated using the RDP Classifier version 2.2 [36].

A non-parametric permutational multivariate ANOVA (PERMANOVA) test [36], implemented in the vegan package for R [37] was used to test the effects of milk production, parity and farm on overall community composition, as measured by weighted UniFrac distance. To test for differences in taxon abundance, a generalized linear model (GLM) was constructed with the statistical package for R. The model used a binomial link function and input data for the model consisted of a two-column matrix containing the number of reads assigned to the taxon (in column 1) and the number of reads assigned to other taxa (in column 2) and *p values* were adjusted using a microbial taxonomy-wide detection rate.

The extent of relationship between bacterial communities was quantified using weighted pairwise UniFrac distances [38]. Communities with small weighted UniFrac distance are composed of phylogenetically similar organisms in similar proportions. To identify bacterial lineages that drive the clustering of microbial communities in each farm, we used the biplot function of the make_emperor.py script to plot the genus-level OTUs in PCoA (Principal Coordinate Analysis) space alongside each Farm. Spearman correlation was used to correlate physiological parameters with OTUs assigned to Succinivibrionaceae and with the abundant genera in Bacteroidetes, Firmicutes and Proteobacteria using R and visualized using the corrplot R package [39].

## Results

### Dietary information

The ingredient composition and the nutritive value of diets fed to both primiparous and multiparous dairy cows for both Farm 12 (M305 = 12,300 kg) and Farm 9 (M305 = 9700 kg) are presented in Table 1. Forage content was lower for cows on Farm 12 and averaged 49.8% of diet DM compared to an average of 56.0% forage on Farm 9. Both farms fed corn silage as their primary forage. Farm 12 fed 77.5% (primiparous) and 71.2% (multiparous) of the forage as corn silage, and 22.6% (primiparous) and 25.9% (multiparous) of the forage as alfalfa silage. Additionally, 2.9% of the forage was grass hay for the multiparous cows only on Farm 12. In contrast, Farm 9 fed 85.0% (primiparous) and 81.9% (multiparous) of the forage as corn silage and the remaining forage 15.0% (primiparous) and 18.1% (multiparous) was triticale silage. Carbohydrates fed as grains consisted of fine ground corn and wheat middlings on Farm 12 (combined, 26.2% DM), while fine ground corn, soybean hulls, corn distillers and citrus pulp were included in the diets for Farm 9 (combined, 25.3% DM). However, the fine ground corn averaged 15.0% DM on Farm 9 and was higher on Farm 12 at 18.2% DM for primiparous cows and 20.6% DM for multiparous cows (Table 1). Analyzed TMR samples indicated the starch content of the multiparous cows on Farm 12 was the highest at 32.1%, compared to the range of the other production groups of 26.1% to 29.2% dietary starch. Subsequently, NDF was higher (31.0%) on Farm 9 than Farm 12 (28.5%) and NFC was lower (42.8%) on Farm 9 than Farm 12 (44.3%). Sugars were similar for all production and parity groups with the exception of the multiparous cows on Farm 9 which had the highest sugar content of 5.4% DM. Dietary fat content tended to be higher in Farm 12 due to dietary fat supplementation compared to Farm 9.

**Table 1:** Composition (% DM) of diets fed to primiparous and multiparous cows on Farm 12 and Farm 9

	Farm 12		Farm 9	
	Primiparous	Multiparous	Primiparous	Multiparous
Ingredient composition, %DM				
Corn Silage^a^	38.9	35.1	47.2	46.2
Alfalfa Silage^a^	11.3	12.8	---	---
Triticale^a^	---	---	8.3	10.2
Grass Hay^a^	---	1.4	---	---
Corn Ground Fine	18.2	20.6	15.2	14.9
Wheat Middlings	8.1	5.4	---	---
Soybean Hulls	---	---	5.5	5.4
Corn Distillers	---	---	3.7	3.6
Citrus Pulp	---	---	1.1	1.1
AminoPlus^b^	3.7	3.8	7.4	7.2
SoyPlus^c^	3.7	3.7	---	---
Canola Meal	9.7	8.9	---	---
Soybean Meal	0.4	1.7	5.5	5.4
Blood Meal	0.5	0.7	1.0	1.0
Urea	---	---	.0.4	0.4
Molasses	2.1	1.6	1.3	1.3
Energy Booster 100^d^	0.4	0.9	---	---
Megalac^e^	0.5	0.6	---	---
Pork-Vegetable Fat	---	---	0.5	0.5
Calcium Carbonate	0.99	1.01	1.09	1.11
Sodium Sesquicarbonate	0.87	0.92	0.74	0.72
Sodium Chloride	0.37	0.38	0.46	0.45
Magnesium Oxide	0.13	0.13	0.18	0.18
Methionine, MFP^f^	0.07	0.05	0.11	0.11
Methionine, SmartamineM^g^	---	0.02	---	---
Mineral-Vitamin Mix^h^	0.06	0.11	0.22	0.22
Rumensin 90^i,j^, mg/kg	150.0	149.2	50.4	50.4
Chromium .propionate 4%,^k,l^ mg/kg	2.60	2.54	1.01	1.01
Chemical composition %DM				
Crude Protein	16.5	16.7	16.2	15.0
Soluble Protein, % CP	31.4	31.2	32.2	28.4
RDP^m^ estimated, % CP	59.1	56.9	60.2	59.4
ADF	18.0	18.9	19.2	19.6
NDF	28.8	28.3	30.6	31.3
NFC^n^	44.0	44.6	42.6	43.1
Sugar	4.5	4.1	4.3	5.4
Starch	27.5	32.1	26.1	29.2
Fat	3.8	3.8	3.4	3.4
Ash	6.9	6.7	7.2	7.2
Ca	.92	.82	.96	.79
P	.48	.48	.34	.33

^a^Forage

^b^Heat treated soybean meal; Ag Processing Incorporated (Omaha, Nebraska)

^c^Extruded soybean meal; West Central Cooperative (Ralston, Iowa)

^d^Rumen inert fat; Milk Specialties (Eden Prairie, Minnesota)

^e^Rumen inert fat; Arm and Hammer Animal Nutrition (Princeton, New Jersey)

^f^Rumen available methionine source; Novus International (Saint Charles, Missouri)

^g^Rumen protected methionine; Adisseo (Alpharetta, Georgia)

^h^Concentration (DM basis); **Farm 12 Primiparous**: 39 mg of Fe/kg, 32,795 mg of Zn/kg, 7695 mg of Cu/kg, 18,091 mg of Mn/kg, 460 mg of Se/kg, 616 mg of Co/kg, 622 mg of I/kg, 3203 KIU of vitamin A/kg, 804 KIU vitamin D/kg, 22 KIU vitamin E/kg; **Farm 12 Multiparous**: 3300 mg of Fe/kg, 21,000 mg of Zn/kg, 5556 mg of Cu/kg, 11,118 mg of Mn/kg, 141 mg of Se/kg, 543 mg of Co/kg, 222 mg of I/kg, 1032 KIU vitamin A/kg, 258 KIU vitamin D/kg, 10 KIU vitamin E/kg; **Farm 9 Primiparous and Multiparous** 4537 mg of Fe/kg, 19,638 mg of Zn/kg, 3724 mg of Cu/kg, 10,157 mg of Mn/kg, 130 mg of Se/kg, 102 mg of Co/kg, 305 mg of I/kg, 1067 KIU of vitamin A/kg, 267 KIU vitamin D/kg, 6 KIU vitamin E/kg;

^i^Elanco Animal Health (Greenfield, Indiana)

^j^Provided 14.6 and 15.0 mg monensin/kg diet DM to Farm 12 primiparous and multiparous cows, respectively, and provided 14.8 and 14.5 mg monensin/kg diet DM to Farm 9 primiparous and multiparous cows, respectively

^k^Kemin (Des Moines, Iowa)

^l^Provided 0.25 and 0.26 mg chromium/kg diet DM to Farm 12 primiparous and multiparous cows, respectively, and provided 0.30 and 0.29 mg chromium/kg diet DM to Farm 9 primiparous and multiparous cows, respectively

^m^Rumen degraded protein estimated using CPM-Dairy software

^n^Non-Fiber Carbohydrate calculated value (100-CP-NDF-Fat-Ash)

### Production information

Test day records from DRMS, Raleigh, NC were extracted for cows sampled on each farm. Production parameters by farm and parity including milk yield (kg/d), milk fat (%) and milk protein (%) are presented in Table 2. A difference in milk yield of 9.5 and 18.1 kg/d was noted between the high and low producing cows for primiparous and multiparous groups, respectively for Farm 12. A difference of 11.7 and 9.7 kg/d was noted between high and low producing cows in the primiparous and multiparous groups, respectively for Farm 9. Milk fat (%) was the lowest (3.20%) in the high producing multiparous cows and was the highest (3.86%) in the low producing multiparous cows from both Farm 9 and Farm 12, most likely associated with the higher milk yields and dilution of milk components. Milk fat (%) was higher for the primiparous cows on Farm 9 (3.73%) as compared to the primiparous cows on Farm 12 (3.32%). Overall for both farms and parity groups, milk fat (%) and protein (%) was higher in low yielding cows compared to high yielding cows for both farms and parity groups.

**Table 2:** Mean with SD of production parameters in high and low yielding cows by parity and farm

	Farm 12				Farm 9			
	Primiparous cows		Multiparous cows		Primiparous cows		Multiparous cows	
	High	Low	High	Low	High	Low	High	Low
	(*n* = 12)	(*n* = 12)	(*n* = 12)	(*n* = 11)	(*n* = 10)	(*n* = 9)	(*n* = 9)	(*n* = 10)
Days in milk	116	109	125	125	128	113	130	132
Milk yield (kg/d)	48.1	38.6	70.7	52.6	37.2	25.5	41.8	32.1
	± 1.3	± 1.2	± 4.3	± 3.3	± 7.1	± 2.7	± 3.4	± 3.4
Fat (%)	3.31	3.34	3.20	3.86	3.64	3.83	3.19	3.86
	± 0.57	± 0.61	± 1.25	± 0.90	± 0.36	± 0.62	± 0.48	± 0.67
Protein (%)	2.85	2.94	2.86	3.23	2.91	2.94	2.87	3.03
	± 0.22	± 0.20	± 0.27	± 0.28	± 0.22	± 0.30	± 0.23	± 0.23

### Sequencing information

Approximately 660,913 reads were analyzed from 85 rumen bacterial communities, with an average of 7775 reads per sample and greater than 3353 reads per sample (Additional file 1: Table S1). Approximately 59,843 OTUs were produced by clustering at 97% sequence similarity. We randomly subsampled (rarified) OTUs to 3353 sequences per sample which produced approximately 42,223 OTUs. From these sequences, 18 bacterial phyla and 73 genera were identified.

### Comparison of bacterial community composition within and between herds

**Fig. 1:**
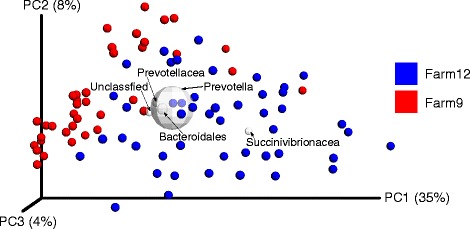
Biplot of weighted UniFrac beta diversity plot, labels for the most 5 abundant genus-level taxa added. The size of the sphere for each taxon is proportional to the mean relative abundance of that taxon across all samples. This plot is created by the QIIME command make_emperor.py

The rumen bacterial communities in the planktonic phase for Farm 12 were significantly different (Adonis: R^2^ = 0.16, *p* < 0.001; Additional file 2: Table S2; Fig. 1) from Farm 9. Bacterial communities in Farm 12 were distantly spaced and spread across the principal coordinate 1 (PC1) whereas, bacterial communities in Farm 9 formed tight clusters on PC2. PC1 was driven by Succinivibrionaceae lineages while the PC2 was driven by *Prevotella*, unclassified Bacteroidales, unclassified Prevotellaceae and an unclassified lineage. Within both herds, the rumen bacterial communities were significantly different by parity (PERMANOVA; *p* < 0.05; Additional file 2: Table S2). However, no such differences were evident by production level within parity group in either herd, with the exception of primiparous cows in Farm 12 (Additional file 2: Table S2).

Irrespective of the herd, Bacteroidetes (75%) and Firmicutes (10–11%) together comprised up to 86% of the bacterial abundance. However, the mean abundance of Proteobacteria (7% vs. 2%) was higher in Farm 12 compared to Farm 9 (Additional file 3: Table S3). Bacteroidetes was predominated by *Prevotella* (48.2%) followed by unclassified Bacteroidales and several other taxa in Farm 9. In Farm 12, *Prevotella* (51.8%) was higher, while unclassified Bacteroidales and RF16 were relatively reduced and S24-7 numerically increased compared to Farm 9 (Additional file 3: Table S3). Among Firmicutes, the majority of taxa decreased in Farm 12 compared to Farm 9, with the exception of *Coprococcus*, unclassified Mogibacteriaceae, *Pseudobutyrivibrio*, *Shuttleworthia* and unclassified Veillonellaceae. Noticeable differences were evident in the total abundance and genera that made up Proteobacteria between both herds. In Farm 12, more than 95% of Proteobacteria was composed of Succinivibrionaceae lineages, whereas in Farm 9, Proteobacteria was comprised of several genera in addition to Succinivibrionaceae lineages (Additional file 3: Table S3).

Differences in the individual bacterial populations are presented for the high and low yielding cows within each parity level in both herds (Table 3). In the high yielding primiparous cows of Farm 12, *Prevotella* from Bacteroidetes was significantly higher compared to the low yielding primiparous cows. The contribution from Firmicutes decreased while that of Proteobacteria increased in the high yielding primiparous cows compared to the low yielding primiparous cows. Members of Succinivibrionaceae lineages were almost doubled (*p* < 0.05) in high versus low yielders in the primiparous cows.

**Table 3:** Mean abundance (%) and SEM of bacterial taxa (identified to the genus level) between low and high yielding cows within parity in Farm 12 and Farm 9

		Primiparous cows			Multiparous cows		
Taxa	Herd	Low	High	*P*-value^‡^	Low	High	*P*-value^‡^
Bacteroidetes (Phylum)							
* Paraprevotellaceae* (family)	Farm12	0.70 ± 0.05	0.57 ± 0.08	*	0.95 ± 0.06	0.82 ± 0.05	
	Farm9	0.85 ± 0.10	0.80 ± 0.05		0.69 ± 0.06	0.58 ± 0.05	
* Bacteroidales* (order)	Farm12	11.54 ± 0.29	10.32 ± 0.30	***	10.54 ± 0.59	9.87 ± 0.32	*
	Farm9	11.28 ± 0.52	12.03 ± 0.59	**	12.43 ± 0.43	11.89 ± 0.38	
* BF311*	Farm12	0.06 ± 0.01	0.08 ± 0.02		0.10 ± 0.04	0.04 ± 0.01	*
	Farm9	0.10 ± 0.03	0.18 ± 0.03	*	0.16 ± 0.03	0.17 ± 0.03	
* CF231*	Farm12	0.70 ± 0.07	0.61 ± 0.07		0.84 ± 0.06	0.73 ± 0.04	
	Farm9	1.16 ± 0.11	1.27 ± 0.12		1.24 ± 0.08	1.06 ± 0.09	
* Prevotella*	Farm12	50.35 ± 1.71	53.39 ± 1.29	***	51.51 ± 1.80	52.05 ± 1.08	
	Farm9	49.55 ± 2.72	45.05 ± 1.77	***	47.60 ± 2.13	50.59 ± 1.74	***
* Prevotellaceae* (family)	Farm12	4.16 ± 0.13	4.22 ± 0.27		3.45 ± 0.25	3.19 ± 0.16	
	Farm9	4.30 ± 0.30	4.36 ± 0.21		5.13 ± 0.27	4.96 ± 0.20	
* RF16* (family)	Farm12	1.14 ± 0.20	0.93 ± 0.10	**	1.07 ± 0.12	1.09 ± 0.09	
	Farm9	2.03 ± 0.28	3.38 ± 0.47	***	1.51 ± 0.24	1.39 ± 0.43	
* S24-7* (family)	Farm12	3.04 ± 0.34	2.34 ± 0.11	***	3.25 ± 0.36	2.99 ± 0.49	
	Farm9	2.21 ± 0.42	2.48 ± 0.27	*	1.70 ± 0.28	1.93 ± 0.31	
* YRC22*	Farm12	0.52 ± 0.06	0.54 ± 0.06		0.54 ± 0.05	0.54 ± 0.05	
	Farm9	0.63 ± 0.05	0.68 ± 0.06		0.85 ± 0.07	0.60 ± 0.06	***
Firmicutes (Phylum)							
* Mogibacteriaceae* (family)	Farm12	0.19 ± 0.03	0.14 ± 0.02		0.27 ± 0.04	0.18 ± 0.03	
	Farm9	0.16 ± 0.05	0.22 ± 0.03		0.11 ± 0.01	0.11 ± 0.02	
* Anaerostipes*	Farm12	NF	NF		0.04 ± 0.01	0.07 ± 0.01	
	Farm9	0.06 ± 0.02	0.09 ± 0.02		NF	NF	
* Anaerovibrio*	Farm12	NF	NF		0.06 ± 0.02	0.04 ± 0.01	
	Farm9	0.04 ± 0.01	0.06 ± 0.01		0.09 ± 0.02	0.08 ± 0.02	
* Asteroleplasma*	Farm12	0.10 ± 0.02	0.09 ± 0.03		0.07 ± 0.02	0.10 ± 0.02	
	Farm9	0.16 ± 0.04	0.10 ± 0.02		0.18 ± 0.03	0.19 ± 0.03	
* Butyrivibrio*	Farm12	0.24 ± 0.04	0.09 ± 0.02	***	0.26 ± 0.05	0.20 ± 0.03	
	Farm9	0.36 ± 0.06	0.24 ± 0.04		0.31 ± 0.05	0.36 ± 0.10	
* Christensenellaceae* (family)	Farm12	0.09 ± 0.02	0.06 ± 0.01		0.07 ± 0.02	0.05 ± 0.01	
	Farm9	0.08 ± 0.02	0.10 ± 0.02		0.08 ± 0.02	0.12 ± 0.02	
* Clostridiales* (order)	Farm12	2.70 ± 0.20	1.89 ± 0.14	***	3.33 ± 0.37	3.25 ± 0.38	
	Farm9	3.07 ± 0.35	3.05 ± 0.16		3.36 ± 0.39	2.98 ± 0.27	
* Coprococcus*	Farm12	0.07 ± 0.01	0.04 ± 0.01		0.18 ± 0.05	0.13 ± 0.02	
	Farm9	0.06 ± 0.01	0.07 ± 0.02		0.05 ± 0.01	0.05 ± 0.01	
* Erysipelotrichaceae* (family)	Farm12	NF	NF		0.03 ± 0.01	0.03 ± 0.01	
	Farm9	0.06 ± 0.01	0.05 ± 0.02		NF	NF	
* Lachnospiraceae* (family)	Farm12	1.31 ± 0.10	1.09 ± 0.12	*	1.53 ± 0.15	1.84 ± 0.15	**
	Farm9	1.48 ± 0.21	1.41 ± 0.10		1.56 ± 0.27	1.46 ± 0.15	
* Moryella*	Farm12	0.06 ± 0.02	0.05 ± 0.01		0.13 ± 0.02	0.10 ± 0.03	
	Farm9	0.13 ± 0.04	0.12 ± 0.02		NF	NF	
* p-75-a5*	Farm12	NF	NF		NF	NF	
	Farm9	0.05 ± 0.01	0.06 ± 0.01		0.06 ± 0.01	0.07 ± 0.02	
* Pseudobutyrivibrio*	Farm12	0.07 ± 0.02	0.05 ± 0.01		0.10 ± 0.03	0.13 ± 0.03	
	Farm9	0.08 ± 0.02	0.05 ± 0.01		0.06 ± 0.02	0.07 ± 0.02	
* RFN20*	Farm12	0.53 ± 0.06	0.42 ± 0.05	*	0.49 ± 0.06	0.38 ± 0.04	
	Farm9	0.79 ± 0.07	0.87 ± 0.09		0.62 ± 0.07	0.65 ± 0.05	
* Ruminococcaceae* (family)	Farm12	1.40 ± 0.14	0.91 ± 0.13	***	1.43 ± 0.17	1.15 ± 0.12	**
	Farm9	1.51 ± 0.25	1.56 ± 0.17		1.24 ± 0.16	1.24 ± 0.12	
* Ruminococcus*	Farm12	0.89 ± 0.13	0.56 ± 0.10	***	0.93 ± 0.09	0.91 ± 0.11	
	Farm9	1.25 ± 0.23	1.17 ± 0.11		1.08 ± 0.26	0.99 ± 0.15	
* Schwartzia*	Farm12	0.06 ± 0.01	0.07 ± 0.02		0.07 ± 0.01	0.07 ± 0.01	
	Farm9	NF	NF		NF	NF	
* Selenomonas*	Farm12	0.14 ± 0.02	0.08 ± 0.02	*	0.17 ± 0.03	0.18 ± 0.03	
	Farm9	0.15 ± 0.03	0.09 ± 0.03		0.20 ± 0.06	0.18 ± 0.03	
* Shuttleworthia*	Farm12	0.08 ± 0.01	0.06 ± 0.01		0.08 ± 0.02	0.10 ± 0.02	
	Farm9	NF	NF		0.05 ± 0.01	0.04 ± 0.01	
* Succiniclasticum*	Farm12	0.17 ± 0.02	0.10 ± 0.02	*	0.20 ± 0.03	0.18 ± 0.03	
	Farm9	0.20 ± 0.02	0.13 ± 0.03		0.13 ± 0.02	0.13 ± 0.03	
* Veillonellaceae* (family)	Farm12	0.66 ± 0.08	0.63 ± 0.08		0.70 ± 0.10	0.70 ± 0.06	
	Farm9	0.55 ± 0.04	0.41 ± 0.05		0.55 ± 0.08	0.55 ± 0.08	
Proteobacteria (Phylum)							
*Alphaproteobacteria* (class)	Farm12	0.07 ± 0.01	0.06 ± 0.01		0.05 ± 0.02	0.08 ± 0.01	
	Farm9	0.14 ± 0.04	0.15 ± 0.03		0.08 ± 0.03	0.07 ± 0.02	
* Deltaproteobacteria* (class)	Farm12	NF	NF		NF	NF	
	Farm9	0.03 ± 0.01	0.05 ± 0.01		0.03 ± 0.01	0.05 ± 0.01	
* RF32* (order)	Farm12	0.11 ± 0.02	0.11 ± 0.02		0.15 ± 0.03	0.09 ± 0.02	*
	Farm9	0.16 ± 0.05	0.18 ± 0.02		0.11 ± 0.03	0.11 ± 0.03	
* Ruminobacter*	Farm12	NF	NF		NF	NF	
	Farm9	0.05 ± 0.02	0.09 ± 0.02		NF	NF	
* Succinivibrionaceae* (family)	Farm12	4.48 ± 0.76	8.11 ± 2.05	***	5.92 ± 1.80	8.02 ± 1.45	***
	Farm9	1.41 ± 0.28	2.68 ± 0.92	***	0.85 ± 0.32	0.66 ± 0.30	*

^**‡**^ *P*-value comparing bacterial abundance of high and low milk production within parity

The magnitude of the P-value (*** *P*<0.001; ** *P*<0.01; * *P*<0.05)

*NF* Not found

In the high yielding multiparous cows of Farm 12, the contribution from Bacteroidetes was lower compared to the low yielding multiparous cows*,* while several Firmicutes lineages increased, unlike what was observed in the primiparous cows. Distinct differences in Proteobacteria populations were not evident between high and low yielding multiparous cows, except for Succinivibrionaceae lineages which were increased in the high yielders (*p* < 0.05). No evident patterns were observed between high and low yielding cows in Farm 9.

## Diversity of Succinivibrionaceae

**Fig. 2:**
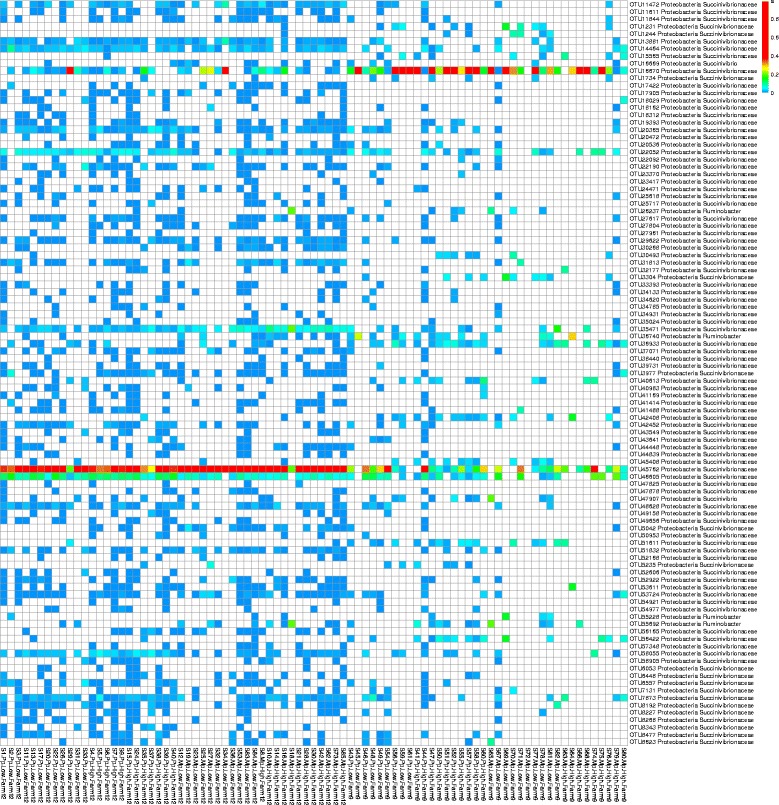
Heatmap showing the distribution of OTUs assigned to Succinivibrionaceae family in dairy cows in Farm 12 and Farm 9

As Succinivibrionaceae lineages were abundant in Farm 12 and were found to be higher (*p* < 0.05) in high yielding primiparous and multiparous cows, we performed additional analysis on the sequences that were annotated to Succinivibrionaceae lineages. Across all samples, we identified a total of 326 OTUs that were assigned to this bacterial family (Fig. 2). Of the 326 OTUs, 143 OTUs were common to both farms. About 141 were unique to Farm 12 and 42 OTUs were identified only in Farm 9. In Farm 12, OTU45762 was found in all samples and comprised 35% of Succinivibrionaceae abundance. In contrast, this OTU was not detected in a majority of samples in Farm 9. Instead OTU16670 was found to be the most abundant OTU in Farm 9, but was less than 5% in Farm 12. Similar patterns were observed for a majority of Succinivibrionaceae OTUs, where the OTUs that were abundant in Farm 12 contributed only a small portion in Farm 9 and vice versa. These results indicate the diversity among Succinivibrionaceae OTUs in Farm 12 is different compared to that of Farm 9.

### Correlations between rumen bacterial populations and production traits

**Fig. 3:**
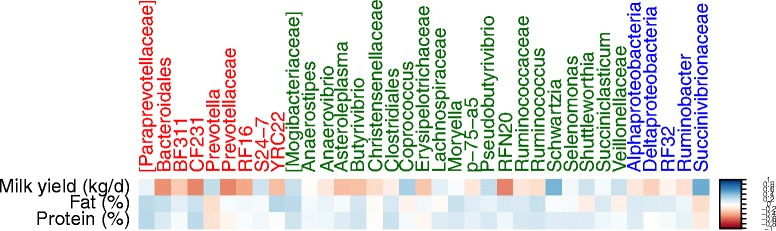
Correlation between production variables and abundant ruminal bacterial genera. The *scale colors* denote whether the correlation is positive (closer to 1, *blue squares*) or negative (closer to −1, *red squares*) between the taxa and the production parameters. The taxa colors *red*, *dark green* and *blue* denotes the genus of Bacteroidetes, Firmicutes and Proteobacteria respectively

To investigate the relationship between bacterial populations and production traits, we performed a Spearman correlation test for the most abundant taxa within Bacteroidetes, Firmicutes and Proteobacteria (Fig. 3). In general, bacterial taxa that were positively correlated with milk yield, showed negative correlations with fat and protein content in milk. *Prevotella* and *S24-7* from Bacteroidetes, and Succinivibrionaceae lineages from Proteobacteria were positively correlated with milk yield. All taxa except Christensenellaceae, *Coprococcus*, Erysipelotrichaceae, Lachnospiraceae, *Shuttleworthia* and Veillonellaceae lineages from Firmicutes (Fig. 3) showed positive correlations with milk fat and protein content.

As the diversity of Succinivibrionaceae was different between farms, we performed correlations between the most abundant Succinivibrionaceae OTUs and production variables in this study (Additional file 4: Figure S1). The OTUs selected for this analysis were OTUs 45,762, 46,605, 22,052, 35,471, 14,464, 58,055, 7873, 20,365, and 11,472) from Farm 12 and the only abundant OTU (16670) from Farm 9. Interestingly, we found that all Succinivibrionaceae OTUs from Farm 12 were found to be positively correlated to milk yield and negatively correlated with fat and protein content, whereas OTU16670 from Farm 9 showed the opposite trend.

## Discussion

In the Pennsylvania dairy sector, average annual milk production per cow (M305) is about 10,000 kg. In this study, Farm 12 (M305 = 12,300 kg) represented the top 5% of Pennsylvania dairy herds and Farm 9 (M305 = 9700 kg) represented the average dairy herd in Pennsylvania [40]. There are many sources to account for variation in milk yield between dairy farms. Genetics accounts for approximately 25% of the variation [41] and management factors, including cow comfort, milking frequency, ration formulation and feeding management contribute to the remaining 75% [42]. While animal genetics, nutrition and management can greatly influence milk production differences among herds, our objective was to investigate how diet impacts microbiota and how rumen bacterial community compositions differ in dairy cows with varying milk production. To accomplish our goal and to obtain large numbers of cows varying in parity and production, we selected two commercial dairy herds, knowing that we would not be able to control the many aspects of herd variation that influence milk yield. We used the stomach tube method for rumen sample collection, realizing only the planktonic associated microbiota is typically retrieved, however this approach allowed us to sample large groups of cows across herds. To this end, we sampled rumen fluid from higher and lower yielding dairy cows across two farms in Pennsylvania and investigated the bacterial diversity using Ion Torrent (PGM) sequencing. The salient findings from this study are that each farm had a unique bacterial profile and the rumen bacterial composition differed substantially between the herds. The inter-herd differences were much greater than intra-herd differences in their rumen bacterial composition, which was not surprising due to large differences in dietary composition between the herds. This study identified the presence of specialist rumen bacteria including Succinivibrionaceae lineages, *Coprococcus* and S24-7, which were associated with higher milk yields in Farm 12.

## Rumen bacterial community composition

Across both farms in this study, Bacteroidetes, mostly comprised of *Prevotella*, Bacteroidales, and Prevotellaceae lineage, and Firmicutes, mainly comprised of Clostridiales*,* Lachnospiraceae*,* and Ruminococcaceae lineages constituted 75% and 10 to 11% of the rumen bacterial composition, respectively. The abundance of Bacteroidetes and Firmicutes in the rumen of dairy cows is a common finding reported by several authors [14–16]. However, the proportion of Bacteroidetes observed in this study was much higher compared to the above reports, but similar to our previous findings [17, 18]. Differences among reports can be attributed to differences in dietary composition, sampling times and methodologies employed in rumen bacterial diversity analysis [43].

Previous reports [15, 16] indicate Proteobacteria accounts for a very small proportion (1–5%) of the rumen bacterial population. Compared to these reports [15, 16], the contribution from Proteobacteria in Farm 12 was higher than expected at 7%, but on the lower end of reported ranges in Farm 9, where Proteobacteria comprised only 2%. Notably, in Farm 12, Succinivibrionaceae lineages represented 97% of the Proteobacteria. The proportions of Bacteroidetes, Firmicutes and Proteobacteria in dairy cows from Farm 9 were similar to our earlier reports from the same herd [17, 18]. This consistency in findings on Farm 9 suggests that the rumen bacterial profiles remain fairly stable for a dairy herd under similar management and dietary regimen.

## Linking diets and rumen bacterial communities

The feed ingredients used in the TMR for Farm 9 and Farm 12 were different. Differences in dietary composition were well reflected in the inter-herd differences in rumen bacterial community composition, which may have contributed to differences in rumen digestion and influenced milk yields between herds. The rumen bacterial communities in Farm 12 were diverse and were driven by *Prevotella,* Bacteroidales, unclassified Prevotellaceae and Succinivibrionaceae, while in Farm 9 there was little variation within rumen bacterial communities, driven mostly by *Prevotella*, Bacteroidales and unclassified Prevotellaceae.

Rumen bacteria with a known function such as S24-7 for butyrate production [44], *Coprococcus* for propionate and butyrate production [22] and Succinivibrionaceae for succinate production [45] were found to be greater in abundance in Farm 12. Notably, *Schwartzia*, a genus from Firmicutes reported to utilize only succinic acid [46] was detected only in Farm 12. These bacteria with specific functions were in greater relative abundance on Farm 12 and observed to be positively correlated with milk production. Recently it was shown that both *Coprococcus* and Succinivibrionaceae were positively correlated with gross feed efficiency in dairy cows [22]. Similarly, Succinivibrionaceae also compete with methanogens for hydrogen required to make succinate, a precursor for propionate [47]. Succinivibrionaceae has roles in mitigating methane production and in producing propionate to supply energy to the host for tissue metabolism [45]. One plausible mechanism for increased milk yield in Farm 12 could be Succinivibrionaceae converting succinate to propionate which is metabolized in the liver to glucose, a precursor for lactose synthesis regulating milk volume [48].

It is well known that the diet fed to a ruminant is a major driver in determining the composition of microbial communities in the rumen [19, 41, 42]. In this study we attribute the inter-herd differences in rumen bacterial composition to dietary factors, particularly forage %DM, forage type, and amount of corn grain and types of byproducts fed on the farms. Forage was fed at a higher %DM on Farm 9 (56%), however, corn silage was the major forage fed on both farms. The starch in properly ensiled corn silage is generally readily available in the rumen due to the moisture and softness of the kernel. However, starch availability in the rumen is also dependent upon maturity and processing of the corn kernel at harvest. Starch from corn silage on Farm 12 may have been more available to rumen microbes due to the use of more sophisticated self-propelled harvesting and kernel processing equipment, allowing kernels to be pulverized completely. Farm 9 used a pull type forage harvester and built-in processor producing kernels that were only knicked or broken into small visible pieces, possibly limiting rumen available starch. In addition to corn silage, Farm 12 fed alfalfa silage to complement their forage base. Alfalfa silage, when fed to steers, showed an increased abundance of Bacteroidetes and Proteobacteria as compared to Sainfoin silage [49]. Further, in experiments that involved feeding a combination of corn silage (33% DM) and alfalfa silage (25% DM), rumen Succinovibrionaceae was detected at 4% in the liquid and 6% in the solid fraction [15]. When corn silage comprised 60% DM and alfalfa silage 9% DM, rumen Succinivibrionaceae was reported at 4% in dairy cows [15]. Similar to these findings, the combination of corn silage and alfalfa silage fed on Farm 12 may have contributed to the higher abundance of rumen Succinivibrionaceae at 4–8%. Succinivibrionaceae OTUs were also more diverse and positively correlated with milk yield in Farm 12 compared to Farm 9, where Succinivibrionaceae OTUs associated with milk production were not detected. Triticale (a hybrid of rye and wheat) silage was fed with corn silage on Farm 9. Triticale is a grass forage and by nature has higher NDF values than legumes harvested at the same relative maturities. Triticale silage had higher NDF concentrations compared to barley and oat silages and resulted in higher molar proportions of acetate and lower molar proportions of butyrate in the rumen compared to barley silage [50]. Congruent to these findings, the NDF concentration on Farm 9 (31.0%) was relatively higher compared to Farm 12 (28.5%). Higher forage %DM and relatively higher NDF content may have contributed to the relative higher concentrations of Fibrobacter and Firmicutes [18, 51, 52] on farm 9 compared to farm 12. Further studies are required to investigate the extent of fermentability of corn silage alone and in combination with other legume and cereal silages on the rumen microbial populations, as these forages form a major component (50 to 70%) in lactating dairy cow diets [53]. Such studies will give insight into dietary-microbial interactions to enhance milk yields in dairy cows.

In addition to forages, non-forage fiber byproducts were fed on both farms. Wheat middlings were the only byproduct fed on Farm 12. In general, wheat middlings contain the highest concentration of starch (19%) compared to many other common byproducts fed to dairy cows. Farm 9 fed corn distillers (12% starch), soyhulls, and citrus pulp, both containing less than 5% starch. In addition, the mean proportion of ground corn (19.4%) in Farm 12 TMR was about 4.3% units greater than that of Farm 9 (15.1%), suggesting increased rumen starch availability on Farm 12. We speculate an increase in rumen Succinivibrionaceae may be associated with greater starch availability in the rumen of high yielding dairy cows in Farm 12. High-grain diets appear to favor the growth of these bacterial populations [45, 54], which agrees with the findings of this study, where corn grain was fed in higher amounts on Farm 12. In addition to higher concentrations of starch, processing of grain has a major impact on the rumen availability of starch, and thus can influence the populations of Succinivibrionaceae in the rumen as revealed by the findings of (Shipp et al, Effects of corn processing method and dietary inclusion of wet distillers grains with soluble on rumen microbial dynamics in finishing steers Ginger, submitted), where the authors observed that Succinivibrionaceae was doubled when finishing beef cattle were fed steam flaked corn compared to dry rolled corn. Again, supporting the increase in rumen Succinivibrionaceae may be associated with greater starch availability in the rumen of high yielding dairy cows in Farm 12. The highest dietary starch concentration in this study (32.1%) was in the multiparous cows on Farm 12, which was also the group with the highest level of milk production. Additionally, DMI increases with the amount of milk produced [3, 53], suggesting cows on Farm 12 ate more TMR DM compared to Farm 9, thus consuming greater amounts of dietary starch to enhance the production of VFA and microbial protein for milk production.

Identification of specialized rumen bacteria is needed for improving productivity of dairy cows [55], and the findings of Pope et al. [45] have created renewed interest in Succinivibrionaceae among the scientific community. Several papers have been published recently concerning this bacterial population [47, 56, 57]. The abundance of Succinivibrionaceae lineages in Farm 12 is noteworthy, particularly the greater abundance of this bacterial population in the high yielding cows in both parity groups. Identification of dietary factors including forage varieties, starch concentration and processing methods can favor the growth of Succinivibrionaceae and other specialist bacteria in the rumen that may positively impact digestion and metabolism of feed substrates to ultimately improve milk production in dairy cows.

## Conclusion

This study compared the rumen bacterial populations between two dairy herds differing by 2600 kg/cow in annual milk production. It can be concluded that within-herd differences are small compared to between-herd differences in the composition of rumen bacterial populations. In this study we attribute inter-herd differences in rumen bacterial composition to dietary factors, particularly forage %DM, forage type, and amount of corn grain and byproducts fed on the farms. The distinct abundance of Succinivibrionaceae lineages in Farm 12 was enlightening, particularly the greater abundance of this bacterial population in the high yielding cows in both parity groups. Our study suggests the growth of Succinivibrionaceae linages may have been associated with greater starch availability in the rumen, where corn grain was fed in higher amounts on Farm 12 and corn silage starch may have also been more readily available due to better kernel processing. Controlled studies are needed to more fully understand the impact of both NDF and starch components in corn silage and other forages on rumen microbial populations. Specific selection of NFC sources including starch, sugars, and non-forage fiber sources to compliment forage inputs will give additional insight into dietary microbial interactions important in improving milk yields on dairy farms. Identification of specialized rumen bacteria in dairy cows capable of improving nutrient utilization and feed conversion are needed to continually improve milk production and feed efficiency on dairy farms. Interactions between diet, rumen microbial and fermentation dynamics, and milk yield and components will expand our knowledge to benefit the dairy industry at large.

## Additional files

Additional file 1: Table S1. Sequencing details and production data for samples from cows on Farms 12 and 9. (DOCX 30 kb)

Additional file 2: Table S2. Multivariate permutational analysis (PERMANOVA) for differences in bacterial communities with regard to milk production and parity, both within and between Farm 12 and Farm 9. (DOCX 15 kb)

Additional file 3: Table S3. Bacterial abundance (%) at phylum, family and genus level for low and high yielding cows within parity in Farm 12 and Farm 9. (DOCX 53 kb)

Additional file 4: Figure S1. Spearman correlation between milk production parameters and most abundant Succinivibrionaceae OTUs across rumen samples. The scale colors denote whether the correlation is positive (closer to 1, blue squares) or negative (closer to −1, red squares) between the Succinivibrionaceae OTU and the milk production parameters. (PDF 12 kb)

## Abbreviations

DIM: Days in milk production
DM: Dry matter
DMI: Dry matter intake
DRMS: Dairy Record Management Systems
FC: Fiber carbohydrates
GLM: generalized linear model
NDF: Neutral detergent fiber
NFC: Non-fiber carbohydrate
OTU: Operational taxonomic unit
PGM: Personnel genome machine
QIIME: Quantitative insights into microbial ecology
TMR: Total mixed rations
