# Supplementary material for: Comparison of rumen bacterial communities in dairy herds of different production
Source: BMC Microbiol. 2017 Aug 30;17:190. doi: 10.1186/s12866-017-1098-z (PMC5577838; doi:10.1186/s12866-017-1098-z)
Supplement: Supplementary file 1 — Sequencing details and production data for samples from cows on Farms 12 and 9. (DOCX 30 kb) [file 12866_2017_1098_MOESM1_ESM.docx]

**Table S1**: Sequencing details and production data for samples from cows on Farms 12 and 9.

| **Sample**  **ID** | **Cow age** | **Milk**  **production** | **Farm** | **Lactation**  **Number** | **Quality**  **filtered**  **reads** | **Milk yield**  **(kg/d)** | **Fat**  **(%)** | **Protein**  **(%)** | **DIM** |
| --- | --- | --- | --- | --- | --- | --- | --- | --- | --- |
| S1 | Primiparous cows | Low | Farm 12 | L1 | 5985 | 38.6 | 3.0 | 3.0 | 114 |
| S2 | Primiparous cows | Low | Farm 12 | L1 | 7124 | 38.6 | 3.5 | 3.0 | 140 |
| S3 | Primiparous cows | Low | Farm 12 | L1 | 6512 | 38.6 | 3.4 | 2.9 | 80 |
| S4 | Primiparous cows | High | Farm 12 | L1 | 6292 | 50.0 | 3.5 | 2.8 | 110 |
| S5 | Primiparous cows | High | Farm 12 | L1 | 7596 | 47.3 | 2.6 | 2.9 | 133 |
| S6 | Primiparous cows | High | Farm 12 | L1 | 7881 | 48.6 | 4.0 | 3.3 | 145 |
| S7 | Primiparous cows | High | Farm 12 | L1 | 6784 | 50.0 | 2.5 | 2.6 | 91 |
| S8 | Primiparous cows | High | Farm 12 | L1 | 8458 | 50.5 | 3.5 | 2.6 | 79 |
| S9 | Primiparous cows | Low | Farm 12 | L1 | 7191 | 39.5 | 3.0 | 2.9 | 93 |
| S10 | Primiparous cows | Low | Farm 12 | L1 | 6901 | 39.5 | 3.6 | 2.9 | 121 |
| S11 | Primiparous cows | High | Farm 12 | L1 | 6056 | 47.3 | 4.1 | 2.9 | 123 |
| S12 | Primiparous cows | Low | Farm 12 | L1 | 6138 | 36.8 | 3.8 | 3.1 | 135 |
| S13 | Primiparous cows | Low | Farm 12 | L1 | 7232 | 39.1 | 2.7 | 2.7 | 85 |
| S14 | Primiparous cows | Low | Farm 12 | L1 | 5162 | 38.2 | 3.0 | 2.8 | 84 |
| S15 | Primiparous cows | High | Farm 12 | L1 | 4249 | 47.3 | 3.2 | 2.9 | 126 |
| S16 | Primiparous cows | Low | Farm 12 | L1 | 8094 | 40.0 | 2.7 | 2.9 | 111 |
| S17 | Primiparous cows | Low | Farm 12 | L1 | 6041 | 35.9 | 2.7 | 2.6 | 80 |
| S18 | Primiparous cows | Low | Farm 12 | L1 | 8769 | 39.5 | 4.6 | 3.3 | 123 |
| S19 | Primiparous cows | Low | Farm 12 | L1 | 5253 | 39.5 | 4.1 | 3.2 | 143 |
| S20 | Primiparous cows | High | Farm 12 | L1 | 6779 | 47.3 | 3.7 | 2.9 | 131 |
| S21 | Primiparous cows | High | Farm 12 | L1 | 9548 | 48.2 | 3.6 | 3.0 | 126 |
| S22 | Primiparous cows | High | Farm 12 | L1 | 8250 | 47.3 | 3.4 | 3.0 | 121 |
| S23 | Primiparous cows | High | Farm 12 | L1 | 8182 | 47.3 | 2.3 | 2.5 | 93 |
| S24 | Primiparous cows | High | Farm 12 | L1 | 5892 | 47.3 | 3.3 | 2.8 | 118 |
| S25 | Primiparous cows | High | Farm 9 | L1 | 6276 | 34.5 | 3.6 | 3.1 | 158 |
| S26 | Primiparous cows | Low | Farm 9 | L1 | 6508 | 22.7 | 4.1 | 2.6 | 100 |
| S27 | Primiparous cows | High | Farm 9 | L1 | 6261 | 33.6 | 3.0 | 2.6 | 140 |
| S28 | Primiparous cows | Low | Farm 9 | L1 | 7463 | 22.7 | 5.0 | 3.3 | 118 |
| S29 | Primiparous cows | Low | Farm 9 | L1 | 6318 | 30.0 | 3.7 | 2.8 | 92 |
| S30 | Primiparous cows | High | Farm 9 | L1 | 3549 | 38.2 | 4.1 | 3.0 | 144 |
| S31 | Primiparous cows | Low | Farm 9 | L1 | 3412 | 28.2 | 3.5 | 3.2 | 163 |
| S32 | Primiparous cows | Low | Farm 9 | L1 | 5133 | 22.7 | 3.9 | 3.4 | 149 |
| S33 | Primiparous cows | High | Farm 9 | L1 | 3689 | 33.6 | 3.6 | 3.1 | 142 |
| S34 | Primiparous cows | High | Farm 9 | L1 | 3490 | 33.2 | 3.6 | 2.6 | 130 |
| S35 | Primiparous cows | High | Farm 9 | L1 | 3719 | 40.9 | 3.7 | 2.9 | 127 |
| S36 | Primiparous cows | Low | Farm 9 | L1 | 9176 | 23.6 | 3.4 | 2.7 | 89 |
| S37 | Primiparous cows | High | Farm 9 | L1 | 4888 | 34.5 | 3.7 | 2.8 | 97 |
| S38 | Primiparous cows | Low | Farm 9 | L1 | 3628 | 25.9 | 2.8 | 2.6 | 109 |
| S39 | Primiparous cows | High | Farm 9 | L1 | 3604 | 56.4 | 4.0 | 3.2 | 108 |
| S40 | Primiparous cows | High | Farm 9 | L1 | 3353 | 32.7 | 3.1 | 2.7 | 106 |
| S41 | Primiparous cows | Low | Farm 9 | L1 | 4141 | 27.3 | 3.8 | 3.0 | 99 |
| S42 | Primiparous cows | High | Farm 9 | L1 | 7548 | 35.5 | 4.0 | 3.1 | 115 |
| S43 | Primiparous cows | Low | Farm 9 | L1 | 4331 | 26.8 | 4.3 | 2.9 | 101 |
| S44 | Multiparous cows | High | Farm 9 | L3 | 6069 | 37.3 | 4.0 | 3.0 | 127 |
| S45 | Multiparous cows | High | Farm 9 | L2 | 5807 | 40.5 | 3.4 | 3.0 | 153 |
| S46 | Multiparous cows | Low | Farm 9 | L3 | 4297 | 33.6 | 4.4 | 3.2 | 93 |
| S47 | Multiparous cows | High | Farm 9 | L2 | 4102 | 43.6 | 3.3 | 3.1 | 114 |
| S48 | Multiparous cows | Low | Farm 9 | L2 | 10528 | 33.6 | 4.0 | 2.7 | 119 |
| S49 | Multiparous cows | High | Farm 9 | L2 | 5354 | 39.1 | 3.7 | 2.7 | 122 |
| S50 | Multiparous cows | Low | Farm 9 | L4 | 5150 | 32.3 | 5.4 | 3.1 | 163 |
| S51 | Multiparous cows | Low | Farm 9 | L2 | 5714 | 33.6 | 3.7 | 3.1 | 114 |
| S52 | Multiparous cows | Low | Farm 9 | L3 | 8079 | 35.0 | 3.8 | 3.0 | 107 |
| S53 | Multiparous cows | Low | Farm 9 | L2 | 8018 | 35.0 | 3.4 | 2.8 | 98 |
| S54 | Multiparous cows | High | Farm 9 | L2 | 11755 | 46.4 | 2.8 | 2.6 | 119 |
| S55 | Multiparous cows | High | Farm 9 | L2 | 3687 | 42.3 | 2.9 | 2.8 | 121 |
| S56 | Multiparous cows | High | Farm 9 | L2 | 6343 | 38.2 | 3.3 | 3.2 | 113 |
| S57 | Multiparous cows | Low | Farm 9 | L3 | 4922 | 26.4 | 3.9 | 3.3 | 164 |
| S58 | Multiparous cows | Low | Farm 9 | L3 | 5782 | 33.2 | 3.5 | 3.1 | 179 |
| S59 | Multiparous cows | High | Farm 9 | L2 | 7419 | 46.8 | 2.8 | 2.9 | 129 |
| S60 | Multiparous cows | High | Farm 9 | L4 | 4913 | 42.7 | 2.5 | 2.5 | 173 |
| S61 | Multiparous cows | Low | Farm 9 | L2 | 5153 | 33.6 | 2.9 | 2.7 | 135 |
| S62 | Multiparous cows | Low | Farm 9 | L3 | 9386 | 25.5 | 3.6 | 3.3 | 149 |
| S63 | Multiparous cows | High | Farm 12 | L2 | 18512 | 65.0 | 3.3 | 2.8 | 102 |
| S64 | Multiparous cows | High | Farm 12 | L5 | 11465 | 69.5 | 3.2 | 3.3 | 139 |
| S65 | Multiparous cows | High | Farm 12 | L5 | 10010 | 72.7 | 3.6 | 3.0 | 117 |
| S66 | Multiparous cows | Low | Farm 12 | L2 | 10450 | 53.1 | 3.9 | 3.3 | 100 |
| S67 | Multiparous cows | High | Farm 12 | L4 | 10146 | 69.5 | 2.8 | 2.7 | 124 |
| S68 | Multiparous cows | High | Farm 12 | L4 | 11408 | 80.9 | 2.5 | 2.6 | 102 |
| S69 | Multiparous cows | Low | Farm 12 | L4 | 10525 | 53.6 | 5.3 | 3.0 | 105 |
| S70 | Multiparous cows | Low | Farm 12 | L2 | 12630 | 51.8 | 4.0 | 3.5 | 150 |
| S71 | Multiparous cows | High | Farm 12 | L3 | 10647 | 68.2 | 2.8 | 3.3 | 138 |
| S72 | Multiparous cows | High | Farm 12 | L4 | 15971 | 75.9 | 3.2 | 3.0 | 102 |
| S73 | Multiparous cows | Low | Farm 12 | L2 | 13340 | 55.5 | 3.8 | 3.2 | 150 |
| S74 | Multiparous cows | High | Farm 12 | L2 | 12794 | 69.5 | 2.6 | 2.9 | 156 |
| S75 | Multiparous cows | High | Farm 12 | L2 | 10471 | 68.6 | 1.3 | 2.6 | 107 |
| S76 | Multiparous cows | High | Farm 12 | L3 | 12437 | 68.6 | 2.2 | 2.6 | 147 |
| S77 | Multiparous cows | Low | Farm 12 | L4 | 13821 | 45.9 | 2.7 | 2.7 | 120 |
| S78 | Multiparous cows | High | Farm 12 | L3 | 10845 | 73.6 | 6.2 | 2.5 | 118 |
| S79 | Multiparous cows | Low | Farm 12 | L2 | 10980 | 52.3 | 3.9 | 3.4 | 139 |
| S80 | Multiparous cows | Low | Farm 12 | L2 | 3738 | 55.0 | 3.7 | 3.1 | 106 |
| S81 | Multiparous cows | Low | Farm 12 | L2 | 14664 | 50.0 | 5.3 | 3.6 | 100 |
| S82 | Multiparous cows | High | Farm 12 | L4 | 9580 | 68.2 | 4.7 | 3.0 | 144 |
| S83 | Multiparous cows | Low | Farm 12 | L3 | 12738 | 50.5 | 2.3 | 3.1 | 158 |
| S84 | Multiparous cows | Low | Farm 12 | L2 | 12970 | 53.2 | 4.0 | 3.6 | 117 |
| S85 | Multiparous cows | Low | Farm 12 | L2 | 15437 | 58.6 | 3.6 | 3.0 | 134 |
