# Supplementary material for: Comparison of rumen bacterial communities in dairy herds of different production
Source: BMC Microbiol. 2017 Aug 30;17:190. doi: 10.1186/s12866-017-1098-z (PMC5577838; doi:10.1186/s12866-017-1098-z)
Supplement: Supplementary file 2 — Multivariate permutational analysis (PERMANOVA) for differences in bacterial communities with regard to milk production and parity, both within and between Farm 12 and Farm 9. (DOCX 15 kb) [file 12866_2017_1098_MOESM2_ESM.docx]

**Table S2:** Multivariate permutational analysis (PERMANOVA) for differences in bacterial communities with regard to milk production and parity, both within and between Farm 12 and Farm 9

| PERMANOVA | | |
| --- | --- | --- |
|  | Weighted UniFrac distances | |
|  | R^2^ | P-value |
| Farm12 vs Farm 9 |  |  |
| Farm (Farm12 vs Farm 9) | 0.16964 | 0.001 |
| Milk production (Low vs High) | 0.01260 | 0.328 |
| Parity ( Primiparous vs Multiparous ) | 0.02447 | 0.047 |
|  |  |  |
| Farm12 |  |  |
| Milk production (Low vs High) | 0.04400 | 0.042 |
| Parity ( Primiparous vs Multiparous ) | 0.05900 | 0.017 |
|  |  |  |
| Farm 9 |  |  |
| Milk production (Low vs High) | 0.02500 | 0.472 |
| Parity ( Primiparous vs Multiparous ) | 0.06300 | 0.009 |
|  |  |  |
| Farm12 Primiparous |  |  |
| Low vs High | 0.09824 | 0.032 |
| Farm12 Multiparous |  |  |
| Low vs High | 0.03457 | 0.633 |
| Farm 9 Primiparous |  |  |
| Low vs High | 0.05806 | 0.297 |
| Farm 9 Multiparous |  |  |
| Low vs High | 0.05060 | 0.499 |
