# Supplementary material for: Comparison of rumen bacterial communities in dairy herds of different production
Source: BMC Microbiol. 2017 Aug 30;17:190. doi: 10.1186/s12866-017-1098-z (PMC5577838; doi:10.1186/s12866-017-1098-z)
Supplement: Supplementary file 3 — Bacterial abundance (%) at phylum, family and genus level for low and high yielding cows within parity in Farm 12 and Farm 9. (DOCX 53 kb) [file 12866_2017_1098_MOESM3_ESM.docx]

**Table S3:** Bacterial abundance (%) at phylum, family and genus level for low and high yielding cows within parity in Farm 12 and Farm 9

**Phylum level**

|  |  |  | **Primiparous cows** | | | **Multiparous cows** | | |
| --- | --- | --- | --- | --- | --- | --- | --- | --- |
|  | **Phylum** | **Avg^†^** | **Low** | **High** | **P-value^‡^** | **Low** | **High** | **P-value^‡^** |
| Farm 12 | Bacteroidetes | 74.69 | 75.36 | 75.52 |  | 74.53 | 73.35 | *** |
|  | Cyanobacteria | 0.56 | 0.34 | 0.41 |  | 0.73 | 0.76 |  |
|  | Fibrobacteres | 0.74 | 0.67 | 0.63 |  | 0.81 | 0.83 |  |
|  | Firmicutes | 9.73 | 9.88 | 7.34 | *** | 11.10 | 10.61 |  |
|  | Proteobacteria | 7.07 | 4.88 | 8.52 | *** | 6.40 | 8.48 | *** |
|  | Spirochaetes | 0.37 | 0.33 | 0.25 |  | 0.47 | 0.45 |  |
|  | SR1 | 0.17 | 0.09 | 0.12 |  | 0.21 | 0.24 |  |
|  | Tenericutes | 0.64 | 0.69 | 0.49 | *** | 0.70 | 0.67 |  |
|  | TM7 | 0.51 | 0.54 | 0.44 |  | 0.54 | 0.51 |  |
|  | WPS-2 | 0.07 | 0.11 | 0.07 |  | 0.07 | 0.03 |  |
|  |  |  | **Primiparous cows** | | | **Multiparous cows** | | |
|  | **Phylum** | **Avg^†^** | **Low** | **High** | **P-value^‡^** | **Low** | **High** | **P-value^‡^** |
| Farm 9 | Bacteroidetes | 74.60 | 74.79 | 73.31 | *** | 74.30 | 76.00 | *** |
|  | Cyanobacteria | 0.81 | 0.61 | 0.69 |  | 0.98 | 0.97 |  |
|  | Fibrobacteres | 1.11 | 0.99 | 1.15 |  | 1.19 | 1.08 |  |
|  | Firmicutes | 11.03 | 11.48 | 10.98 |  | 11.06 | 10.62 |  |
|  | Proteobacteria | 2.04 | 2.19 | 3.47 | *** | 1.42 | 1.08 | *** |
|  | Spirochaetes | 0.35 | 0.38 | 0.40 |  | 0.38 | 0.26 | * |
|  | SR1 | 0.16 | 0.18 | 0.12 |  | 0.13 | 0.20 | * |
|  | Tenericutes | 1.01 | 1.08 | 1.24 |  | 0.81 | 0.90 |  |
|  | TM7 | 0.85 | 0.79 | 0.71 |  | 1.01 | 0.88 |  |
|  | WPS-2 | 0.10 | 0.10 | 0.12 |  | 0.06 | 0.11 | * |

**Family level**

|  |  | **Avg^†^** | **Primiparous cows** | | | **Multiparous cows** | | |
| --- | --- | --- | --- | --- | --- | --- | --- | --- |
|  | **Family** |  | **Low** | **High** | **P-value‡** | **Low** | **High** | **P-value^‡^** |
| Farm 12 | Bacteria | 5.29 | 6.86 | 6.03 | *** | 4.32 | 3.94 | * |
|  | Bacteroidetes | 2.38 | 3.11 | 2.36 | *** | 2.10 | 1.95 |  |
|  | Bacteroidetes [Paraprevotellaceae] | 2.03 | 1.94 | 1.75 |  | 2.36 | 2.09 | * |
|  | Bacteroidetes Bacteroidaceae | 0.07 | 0.07 | 0.09 |  | 0.10 | 0.04 | * |
|  | Bacteroidetes Bacteroidales | 10.57 | 11.54 | 10.32 | *** | 10.54 | 9.87 | * |
|  | Bacteroidetes Prevotellaceae | 55.58 | 54.50 | 57.61 | *** | 54.96 | 55.23 |  |
|  | Bacteroidetes RF16 | 1.06 | 1.14 | 0.93 | ** | 1.07 | 1.09 |  |
|  | Bacteroidetes S24-7 | 2.90 | 3.04 | 2.34 | *** | 3.25 | 2.99 |  |
|  | Cyanobacteria YS2 | 0.55 | 0.33 | 0.40 |  | 0.71 | 0.74 |  |
|  | Fibrobacteres Fibrobacteraceae | 0.74 | 0.67 | 0.63 |  | 0.81 | 0.83 |  |
|  | Firmicutes | 0.54 | 0.65 | 0.54 |  | 0.49 | 0.47 |  |
|  | Firmicutes [Mogibacteriaceae] | 0.22 | 0.22 | 0.17 |  | 0.29 | 0.21 |  |
|  | Firmicutes Christensenellaceae | 0.07 | 0.09 | 0.06 |  | 0.07 | 0.05 |  |
|  | Firmicutes Clostridiales | 2.79 | 2.70 | 1.89 | *** | 3.33 | 3.25 |  |
|  | Firmicutes Erysipelotrichaceae | 0.69 | 0.82 | 0.64 | ** | 0.72 | 0.57 | * |
|  | Firmicutes Lachnospiraceae | 2.06 | 1.86 | 1.43 | *** | 2.35 | 2.58 |  |
|  | Firmicutes Ruminococcaceae | 2.07 | 2.33 | 1.49 | *** | 2.40 | 2.09 | * |
|  | Firmicutes Veillonellaceae | 1.26 | 1.16 | 1.11 |  | 1.41 | 1.36 |  |
|  | Proteobacteria | 0.09 | 0.09 | 0.11 |  | 0.08 | 0.07 |  |
|  | Proteobacteria Alphaproteobacteria | 0.06 | 0.07 | 0.06 |  | 0.05 | 0.08 |  |
|  | Proteobacteria RF32 | 0.11 | 0.11 | 0.11 |  | 0.15 | 0.09 |  |
|  | Proteobacteria Succinivibrionaceae | 6.69 | 4.52 | 8.14 | *** | 6.01 | 8.11 | *** |
|  | Spirochaetes Spirochaetaceae | 0.36 | 0.31 | 0.24 |  | 0.45 | 0.42 |  |
|  | SR1 | 0.17 | 0.09 | 0.12 |  | 0.21 | 0.24 |  |
|  | Tenericutes Anaeroplasmataceae | 0.48 | 0.55 | 0.41 | ** | 0.48 | 0.46 |  |
|  | Tenericutes Mollicutes | 0.04 | 0.05 | 0.02 | * | 0.05 | 0.04 |  |
|  | Tenericutes Mycoplasmataceae | 0.07 | 0.06 | 0.04 |  | 0.06 | 0.10 |  |
|  | TM7 F16 | 0.51 | 0.54 | 0.44 |  | 0.54 | 0.51 |  |
|  | Unclassified | 0.07 | 0.11 | 0.07 |  | 0.05 | 0.05 |  |
|  | WPS-2 | 0.07 | 0.11 | 0.07 |  | 0.07 | 0.03 |  |
|  |  |  | **Primiparous cows** | | | **Multiparous cows** | | |
|  | **Family** | **Avg^†^** | **Low** | **High** | **P-value^‡^** | **Low** | **High** | **P-value^‡^** |
| Farm 9 | Bacteria | 7.70 | 7.15 | 7.50 |  | 8.50 | 7.66 | ** |
|  | Bacteroidetes | 2.81 | 2.60 | 2.97 | * | 2.89 | 2.79 |  |
|  | Bacteroidetes [Paraprevotellaceae] | 2.62 | 2.66 | 2.77 |  | 2.80 | 2.25 | *** |
|  | Bacteroidetes Bacteroidaceae | 0.18 | 0.12 | 0.22 | * | 0.19 | 0.19 |  |
|  | Bacteroidetes Bacteroidales | 11.91 | 11.28 | 12.03 | * | 12.43 | 11.89 |  |
|  | Bacteroidetes Prevotellaceae | 52.89 | 53.86 | 49.42 | *** | 52.73 | 55.55 | *** |
|  | Bacteroidetes RF16 | 2.08 | 2.03 | 3.38 | *** | 1.51 | 1.39 |  |
|  | Bacteroidetes S24-7 | 2.08 | 2.21 | 2.48 |  | 1.70 | 1.93 |  |
|  | Cyanobacteria YS2 | 0.79 | 0.60 | 0.65 |  | 0.95 | 0.96 |  |
|  | Fibrobacteres Fibrobacteraceae | 1.11 | 0.99 | 1.15 |  | 1.19 | 1.08 |  |
|  | Firmicutes | 0.75 | 0.77 | 0.65 |  | 0.81 | 0.80 |  |
|  | Firmicutes [Mogibacteriaceae] | 0.18 | 0.18 | 0.27 | * | 0.14 | 0.13 |  |
|  | Firmicutes Christensenellaceae | 0.10 | 0.08 | 0.10 |  | 0.08 | 0.12 |  |
|  | Firmicutes Clostridiales | 3.12 | 3.07 | 3.05 |  | 3.36 | 2.98 | * |
|  | Firmicutes Erysipelotrichaceae | 1.24 | 1.29 | 1.36 |  | 1.11 | 1.22 |  |
|  | Firmicutes Lachnospiraceae | 2.13 | 2.23 | 2.02 |  | 2.15 | 2.12 |  |
|  | Firmicutes Ruminococcaceae | 2.53 | 2.79 | 2.76 |  | 2.34 | 2.25 |  |
|  | Firmicutes Veillonellaceae | 0.90 | 0.95 | 0.70 | ** | 1.00 | 0.95 |  |
|  | Proteobacteria | 0.15 | 0.16 | 0.19 |  | 0.16 | 0.10 |  |
|  | Proteobacteria Alphaproteobacteria | 0.11 | 0.14 | 0.15 |  | 0.08 | 0.07 |  |
|  | Proteobacteria RF32 | 0.14 | 0.16 | 0.18 |  | 0.11 | 0.11 |  |
|  | Proteobacteria Succinivibrionaceae | 1.49 | 1.50 | 2.81 | *** | 0.96 | 0.70 | ** |
|  | Spirochaetes Spirochaetaceae | 0.32 | 0.34 | 0.35 |  | 0.33 | 0.23 |  |
|  | SR1 | 0.16 | 0.18 | 0.12 |  | 0.13 | 0.20 |  |
|  | Tenericutes Anaeroplasmataceae | 0.68 | 0.66 | 0.87 | * | 0.52 | 0.64 |  |
|  | Tenericutes Mollicutes | 0.13 | 0.17 | 0.15 |  | 0.10 | 0.08 |  |
|  | Tenericutes Mycoplasmataceae | 0.13 | 0.16 | 0.15 |  | 0.10 | 0.11 |  |
|  | TM7 F16 | 0.84 | 0.79 | 0.71 |  | 1.01 | 0.88 |  |
|  | Unclassified | 0.11 | 0.13 | 0.11 |  | 0.08 | 0.11 |  |
|  | WPS-2 | 0.10 | 0.10 | 0.12 |  | 0.06 | 0.11 |  |

**Genus level**

|  |  |  | **Primiparous cows** | | | **Multiparous cows** | | |
| --- | --- | --- | --- | --- | --- | --- | --- | --- |
|  | **Genus** | **Avg^†^** | **Low** | **High** | **P-value^‡^** | **Low** | **High** | **P-value^‡^** |
| Farm 12 | Bacteroidetes [Paraprevotellaceae] | 0.76 | 0.70 | 0.57 | * | 0.95 | 0.82 |  |
|  | Bacteroidetes Bacteroidales | 10.57 | 11.54 | 10.32 | *** | 10.54 | 9.87 | * |
|  | Bacteroidetes BF311 | 0.07 | 0.06 | 0.08 |  | 0.10 | 0.04 | * |
|  | Bacteroidetes CF231 | 0.72 | 0.70 | 0.61 |  | 0.84 | 0.73 |  |
|  | Bacteroidetes Prevotella | 51.82 | 50.35 | 53.39 | *** | 51.51 | 52.05 |  |
|  | Bacteroidetes Prevotellaceae | 3.75 | 4.16 | 4.22 |  | 3.45 | 3.19 |  |
|  | Bacteroidetes RF16 | 1.06 | 1.14 | 0.93 | ** | 1.07 | 1.09 |  |
|  | Bacteroidetes S24-7 | 2.90 | 3.04 | 2.34 | *** | 3.25 | 2.99 |  |
|  | Bacteroidetes YRC22 | 0.53 | 0.52 | 0.54 |  | 0.54 | 0.54 |  |
|  | Firmicutes Mogibacteriaceae | 0.20 | 0.19 | 0.14 |  | 0.27 | 0.18 |  |
|  | Firmicutes Anaerostipes | 0.05 | NF | NF |  | 0.04 | 0.07 |  |
|  | Firmicutes Anaerovibrio | 0.05 | NF | NF |  | 0.06 | 0.04 |  |
|  | Firmicutes Asteroleplasma | 0.09 | 0.10 | 0.09 |  | 0.07 | 0.10 |  |
|  | Firmicutes Butyrivibrio | 0.20 | 0.24 | 0.09 | *** | 0.26 | 0.20 |  |
|  | Firmicutes Christensenellaceae | 0.07 | 0.09 | 0.06 |  | 0.07 | 0.05 |  |
|  | Firmicutes Clostridiales | 2.79 | 2.70 | 1.89 | *** | 3.33 | 3.25 |  |
|  | Firmicutes Coprococcus | 0.11 | 0.07 | 0.04 |  | 0.18 | 0.13 |  |
|  | Firmicutes Erysipelotrichaceae | 0.03 | NF | NF |  | 0.03 | 0.03 |  |
|  | Firmicutes Lachnospiraceae | 1.44 | 1.31 | 1.09 | * | 1.53 | 1.84 |  |
|  | Firmicutes Moryella | 0.09 | 0.06 | 0.05 |  | 0.13 | 0.10 | ** |
|  | Firmicutes Pseudobutyrivibrio | 0.09 | 0.07 | 0.05 |  | 0.10 | 0.13 |  |
|  | Firmicutes RFN20 | 0.46 | 0.53 | 0.42 | * | 0.49 | 0.38 |  |
|  | Firmicutes Ruminococcaceae | 1.22 | 1.40 | 0.91 | *** | 1.43 | 1.15 |  |
|  | Firmicutes Ruminococcus | 0.82 | 0.89 | 0.56 | *** | 0.93 | 0.91 | ** |
|  | Firmicutes Schwartzia | 0.07 | 0.06 | 0.07 |  | 0.07 | 0.07 |  |
|  | Firmicutes Selenomonas | 0.14 | 0.14 | 0.08 | * | 0.17 | 0.18 |  |
|  | Firmicutes Shuttleworthia | 0.08 | 0.08 | 0.06 |  | 0.08 | 0.10 |  |
|  | Firmicutes Succiniclasticum | 0.16 | 0.17 | 0.10 | * | 0.20 | 0.18 |  |
|  | Firmicutes Veillonellaceae | 0.67 | 0.66 | 0.63 |  | 0.70 | 0.70 |  |
|  | Proteobacteria Alphaproteobacteria | 0.06 | 0.07 | 0.06 |  | 0.05 | 0.08 |  |
|  | Proteobacteria RF32 | 0.11 | 0.11 | 0.11 |  | 0.15 | 0.09 | * |
|  | Proteobacteria Succinivibrionaceae | 6.63 | 4.48 | 8.11 | *** | 5.92 | 8.02 | *** |
|  |  |  | **Primiparous cows** | | | **Multiparous cows** | | |
|  | **Genus** | **Avg^†^** | **Low** | **High** | **P-value^‡^** | **Low** | **High** | **P-value^‡^** |
| Farm 9 | Bacteroidetes [Paraprevotellaceae] | 0.73 | 0.85 | 0.80 |  | 0.69 | 0.58 |  |
|  | Bacteroidetes Bacteroidales | 11.91 | 11.28 | 12.03 | ** | 12.43 | 11.89 |  |
|  | Bacteroidetes BF311 | 0.15 | 0.10 | 0.18 | * | 0.16 | 0.17 |  |
|  | Bacteroidetes CF231 | 1.18 | 1.16 | 1.27 |  | 1.24 | 1.06 |  |
|  | Bacteroidetes Prevotella | 48.20 | 49.55 | 45.05 | *** | 47.60 | 50.59 | *** |
|  | Bacteroidetes Prevotellaceae | 4.69 | 4.30 | 4.36 |  | 5.13 | 4.96 |  |
|  | Bacteroidetes RF16 | 2.08 | 2.03 | 3.38 | *** | 1.51 | 1.39 |  |
|  | Bacteroidetes S24-7 | 2.08 | 2.21 | 2.48 | * | 1.70 | 1.93 |  |
|  | Bacteroidetes YRC22 | 0.69 | 0.63 | 0.68 |  | 0.85 | 0.60 | *** |
|  | Firmicutes Mogibacteriaceae | 0.15 | 0.16 | 0.22 |  | 0.11 | 0.11 |  |
|  | Firmicutes Anaerostipes | 0.07 | 0.06 | 0.09 |  | NF | NF |  |
|  | Firmicutes Anaerovibrio | 0.07 | 0.04 | 0.06 |  | 0.09 | 0.08 |  |
|  | Firmicutes Asteroleplasma | 0.16 | 0.16 | 0.10 |  | 0.18 | 0.19 |  |
|  | Firmicutes Butyrivibrio | 0.32 | 0.36 | 0.24 |  | 0.31 | 0.36 |  |
|  | Firmicutes Christensenellaceae | 0.10 | 0.08 | 0.10 |  | 0.08 | 0.12 |  |
|  | Firmicutes Clostridiales | 3.12 | 3.07 | 3.05 |  | 3.36 | 2.98 |  |
|  | Firmicutes Coprococcus | 0.06 | 0.06 | 0.07 |  | 0.05 | 0.05 |  |
|  | Firmicutes Erysipelotrichaceae | 0.05 | 0.06 | 0.05 |  | NF | NF |  |
|  | Firmicutes Lachnospiraceae | 1.48 | 1.48 | 1.41 |  | 1.56 | 1.46 |  |
|  | Firmicutes Moryella | 0.13 | 0.13 | 0.12 |  | NF | NF |  |
|  | Firmicutes p-75-a5 | 0.06 | 0.05 | 0.06 |  | 0.06 | 0.07 |  |
|  | Firmicutes Pseudobutyrivibrio | 0.06 | 0.08 | 0.05 |  | 0.06 | 0.07 |  |
|  | Firmicutes RFN20 | 0.74 | 0.79 | 0.87 |  | 0.62 | 0.65 |  |
|  | Firmicutes Ruminococcaceae | 1.39 | 1.51 | 1.56 |  | 1.24 | 1.24 |  |
|  | Firmicutes Ruminococcus | 1.12 | 1.25 | 1.17 |  | 1.08 | 0.99 |  |
|  | Firmicutes Selenomonas | 0.15 | 0.15 | 0.09 |  | 0.20 | 0.18 |  |
|  | Firmicutes Shuttleworthia | 0.05 | NF | NF |  | 0.05 | 0.04 |  |
|  | Firmicutes Succiniclasticum | 0.15 | 0.20 | 0.13 |  | 0.13 | 0.13 |  |
|  | Firmicutes Veillonellaceae | 0.52 | 0.55 | 0.41 |  | 0.55 | 0.55 |  |
|  | Proteobacteria Alphaproteobacteria | 0.11 | 0.14 | 0.15 |  | 0.08 | 0.07 |  |
|  | Proteobacteria Deltaproteobacteria | 0.04 | 0.03 | 0.05 |  | 0.03 | 0.05 | * |
|  | Proteobacteria RF32 | 0.14 | 0.16 | 0.18 |  | 0.11 | 0.11 |  |
|  | Proteobacteria Ruminobacter | 0.07 | 0.05 | 0.09 |  | NF | NF |  |
|  | Proteobacteria Succinivibrionaceae | 1.40 | 1.41 | 2.68 | *** | 0.85 | 0.66 |  |

**^†^** *Mean abundance of bacterial taxa across all samples*

**^‡^** *P-value comparing bacterial abundance of high and low milk production within parity*

*The magnitude of the P-value (*** P<0.001; ** P<0.01; * P<0.05)*

*NF: Not found*
