# Supplementary figures and images for: Comparison of rumen bacterial communities in dairy herds of different production
Source: BMC Microbiol. 2017 Aug 30;17:190. doi: 10.1186/s12866-017-1098-z (PMC5577838; doi:10.1186/s12866-017-1098-z)

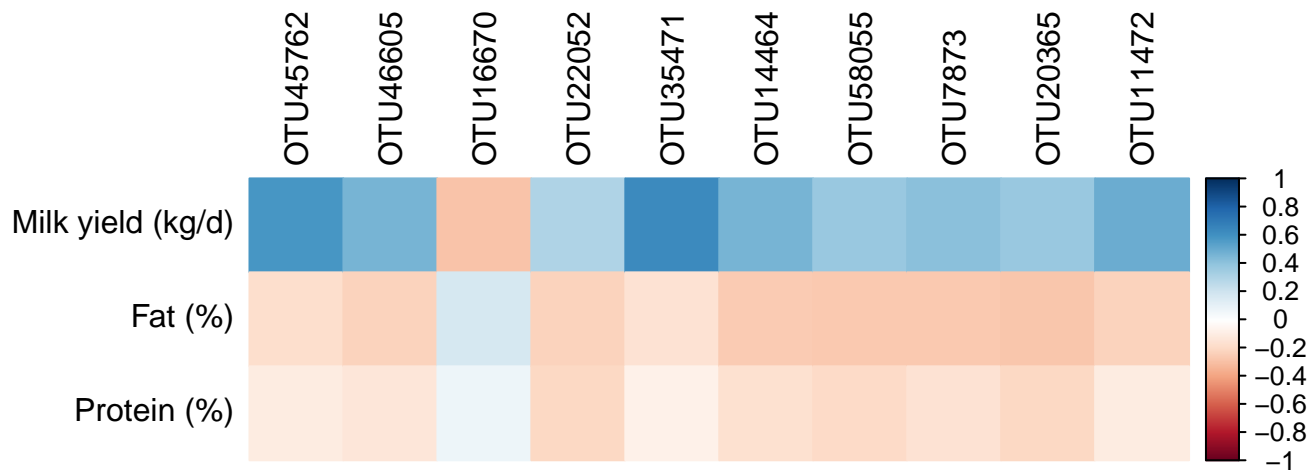

Supplement: Supplementary file 4 — Spearman correlation between milk production parameters and most abundant Succinivibrionaceae OTUs across rumen samples. The scale colors denote whether the correlation is positive (closer to 1, blue squares) or negative (closer to −1, red squares) between the Succinivibrionaceae OTU and the milk production parameters. (PDF 12 kb) [file 12866_2017_1098_MOESM4_ESM.pdf]
